# Architecture of native kinetochores revealed by structural studies utilizing a thermophilic yeast

**DOI:** 10.1016/j.cub.2024.07.036

**Published:** 2024-08-09

**Authors:** Daniel J. Barrero, Sithara S. Wijeratne, Xiaowei Zhao, Grace F. Cunningham, Rui Yan, Christian R. Nelson, Yasuhiro Arimura, Hironori Funabiki, Charles L. Asbury, Zhiheng Yu, Radhika Subramanian, Sue Biggins

**Affiliations:** 1 Howard Hughes Medical Institute, Division of Basic Sciences, Fred Hutchinson Cancer Center, 1100 Fairview Ave. N., Seattle, WA 98109, USA; 2 Molecular and Cellular Biology Program, University of Washington, 1705 NE Pacific Street, Seattle, WA 98195, USA; 3 Department of Molecular Biology, Massachusetts General Hospital, 185 Cambridge Street, Boston, MA 02114, USA; 4 Department of Genetics, Harvard Medical School, 77 Avenue Louis Pasteur, Boston, MA 02115, USA; 5 Howard Hughes Medical Institute Janelia Research Campus, 19700 Helix Drive, Ashburn, VA 20147, USA; 6 The Rockefeller University, 1230 York Ave., New York, NY 10065, USA; 7 Department of Physiology and Biophysics, University of Washington, 1959 NE Pacific Street, Seattle, WA 98195, USA; 8 X (formerly Twitter): @SueBiggins; 9 Lead contact

## Abstract

Eukaryotic chromosome segregation requires kinetochores, multi-megadalton protein machines that assemble on the centromeres of chromosomes and mediate attachments to dynamic spindle microtubules. Kinetochores are built from numerous complexes, and there has been progress in structural studies on recombinant subassemblies. However, there is limited structural information on native kinetochore architecture. To address this, we purified functional, native kinetochores from the thermophilic yeast *Kluyveromyces marxianus* and examined them by electron microscopy (EM), cryoelectron tomography (cryo-ET), and atomic force microscopy (AFM). The kinetochores are extremely large, flexible assemblies that exhibit features consistent with prior models. We assigned kinetochore polarity by visualizing their interactions with microtubules and locating the microtubule binder, Ndc80c. This work shows that isolated kinetochores are more dynamic and complex than what might be anticipated based on the known structures of recombinant subassemblies and provides the foundation to study the global architecture and functions of kinetochores at a structural level.

## In brief

Chromosome segregation requires kinetochores, macromolecular machines that assemble on centromeres. To understand their functions, structural information about kinetochores is critical. Barrero et al. purify native kinetochores from a thermophilic yeast and perform electron microscopy and atomic force microscopy (AFM) to provide insights into kinetochore architecture.

## Graphical Abstract

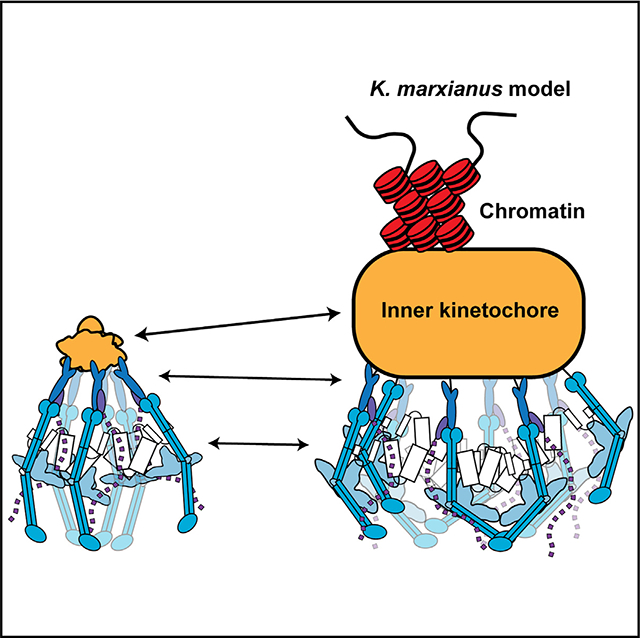

## INTRODUCTION

Eukaryotic cells utilize kinetochores to harness the growth and shortening of spindle microtubules to carry chromosomes toward the spindle poles during cell division.^1–3^ The kinetochore assembles at centromeres, specialized chromosomal regions that are epigenetically specified by the CENP-A histone variant (Cse4 in budding yeast).^4–6^ Multiple copies of kinetochore complexes called the constitutive centromere-associated network (CCAN) assemble on centromeric chromatin to create the inner kinetochore.^7–11^ The CCAN recruits additional outer kinetochore complexes that bind directly to spindle microtubules to complete the link between the chromosome and the microtubule.^12–15^ In addition to their fundamental role in microtubule attachment, kinetochores also serve as a dynamic signaling hub for mitosis, sensing tension, and microtubule attachment as well as creating a scaffold for the spindle assembly checkpoint.^16–20^ A critical step in understanding the diverse functions of kinetochores is to elucidate the structure of the kinetochore.

**Figure 1.**
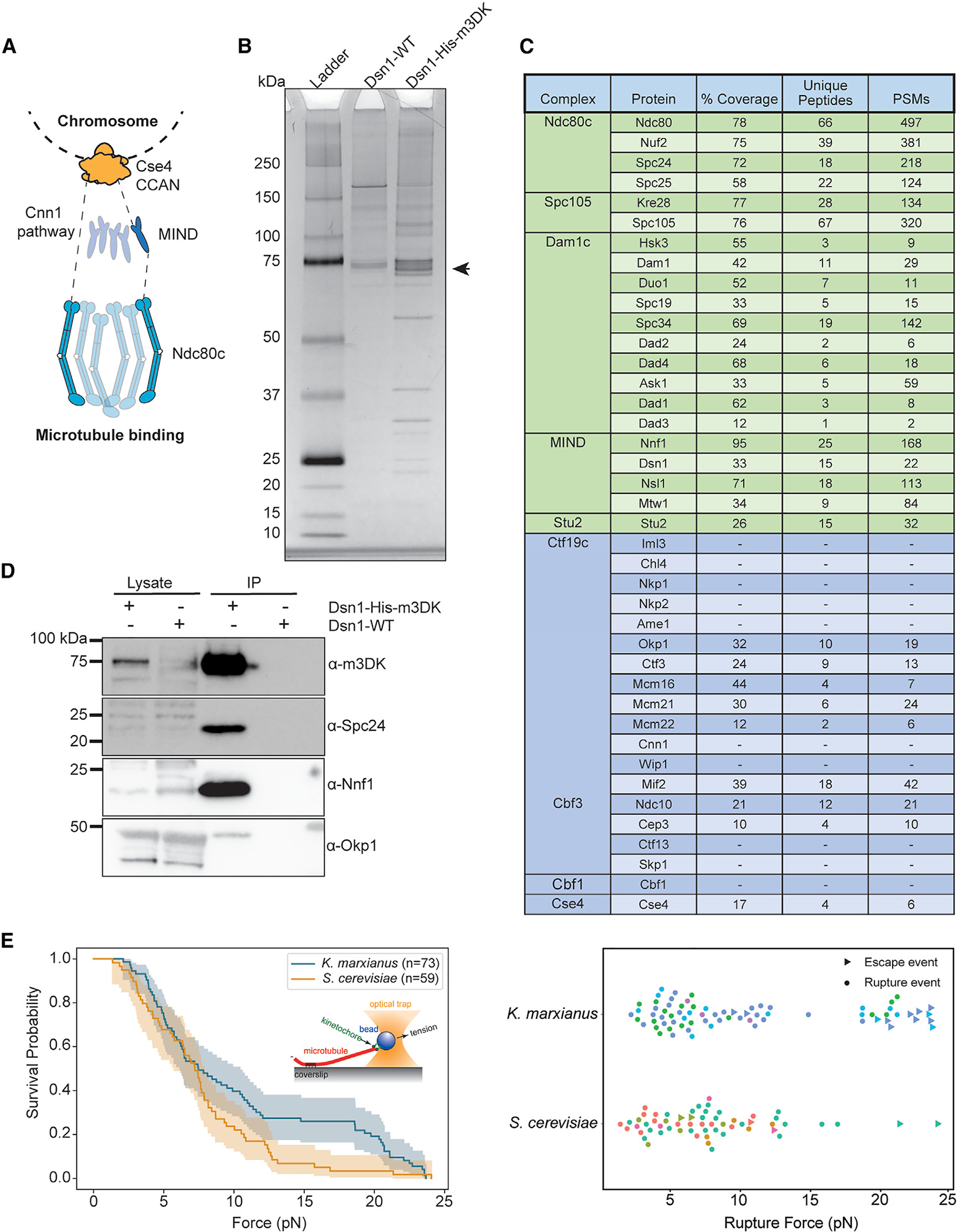
Functional kinetochores can be purified from *K. marxianus* (A) A schematic of kinetochore organization in *S. cerevisiae*. Ndc80c is recruited by either Cnn1 (left) or the MIND complex (right). (B) Kinetochore purifications via Dsn1–6His-3M3DK from an untagged negative control strain (Dsn1 WT; strain SBY17411) or a tagged strain (Dsn1-His-M3DK; strain SBY 18150) were visualized by silver stain. The arrow indicates the position of Dsn1-m3DK, which is inferred from immunoblotting. (C) A table of kinetochore proteins from a representative mass spectrometry experiment of purified *K. marxianus* kinetochores. Rows colored green are outer kinetochore proteins, and rows colored blue are inner kinetochore proteins. Proteins not found in the mass spectrometry data are indicated by dashes. PSM refers to peptide-spectrum matches. (D) Immunoblotting of representative components of the inner and outer kinetochore with the indicated antibodies. Lysate samples were collected before kinetochore purification. Immunoprecipitation (IP) lanes contain purified sample eluted from α-M3DK magnetic beads. (E) Left: survival probability curves of force ramp experiments of *K. marxianus* kinetochores (orange, median = 7.5 pN) and *S. cerevisiae* kinetochores (blue, median = 7.2 pN). Shaded regions represent the 95% confidence intervals. The survival curves differ significantly (*p* = 0.04 by log-rank test). Right: scatterplots of individual rupture force values. Circles represent true ruptures, and triangles represent escape events. Points are colored according to biological replicate. See also Figure S1, Table S1, and Data S1.

Major challenges for kinetochore structural studies are the size, complexity, and dynamics of these machines. Mammalian centromeres span megabases of DNA and contain hundreds of kinetochore proteins that attach to multiple microtubules.^3,8^ By contrast, budding yeasts have point centromeres that contain a single centromeric nucleosome and bind to one microtubule.^4,21–23^ Despite these differences, the major components and functions of kinetochores are generally conserved. Approaches focused on specific kinetochore complexes have yielded structural information and insights into kinetochore complexes and larger assemblies in recent years.^10,24^ Nearly complete yeast and human CCAN complexes, with some including the centromeric nucleosome, have been solved.^25–28^ These structures demonstrate that the 14-member CCAN complex forms a wishbone-like shape, creating a slot in which the centromeric nucleosome can anchor. However, the precise connections between CCAN and the nucleosome have not been visualized^29^ (Figure 1A).

Extensive structural work has also been performed on outer kinetochore complexes. The MIND heterotetramer (Mis12 in humans) that links the microtubule-binding complexes to the inner kinetochore assembles into a Y-shaped structure containing the N termini of its components in the two “heads” of the Y and their C termini located in the tail.^30,31^ The yeast MIND head regions bind to the inner kinetochore yeast proteins Ame1 (CENP-U in humans) and Mif2 (CENP-C in humans).^30,31^ The C termini in turn bind to the Ndc80 complex (Ndc80c), thus completing a continuous connection from the microtubule to the chromosome (Figure 1A). The Ndc80c is a heterotetramer that forms an extended coiled-coil with globular domains at both ends.^32–34^ The globular domains comprised of Spc24 and Spc25 contact the C terminus of the MIND complex, while the globular domains of Nuf2 and, in particular, Ndc80 contact the microtubule.^12,13,35,36^ Ndc80c is also recruited by the inner kinetochore Cnn1 complex (Cnn1c; CENP-TW in humans), which competes with the MIND pathway in yeast^37–39^ (Figure 1A).

Despite progress in solving various subregion structures, it has been difficult to build a complete model of kinetochore architecture from recombinant proteins. The links between the inner and outer kinetochore are made by proteins with long tails that are predicted to be disordered. Indeed, for many structures of kinetochore complexes, it was necessary to remove or reduce flexible elements from the proteins. The available outer kinetochore substructures also lack many proteins and post-translational modifications known to contribute to kinetochore assembly and function.^17,30,40–42^ To address these concerns, structural studies must be performed on native kinetochores to fully understand the spatial relationships and organization of their components.

The only organism from which functional large native kinetochore have been isolated is the budding yeast *Saccharomyces cerevisiae*.^16^ These kinetochores were used in negative stain electron microscopy (EM) experiments; however, the strong tendency for the kinetochores to aggregate and denature on the grids made it difficult to draw conclusions about the architecture.^43^ Likewise, attempts to visualize kinetochores bound to microtubules in vitreous ice offered exciting glimpses of cloud-like masses but did not result in sufficient detail to describe their architecture.^44^ To overcome these limitations, we sought to purify native kinetochores from a thermophilic organism because their adaptation to live at high temperatures facilitates structural biology.^45–47^

Here, we report the purification of functional native kinetochores from the thermophilic yeast *Kluyveromyces marxianus*. These kinetochores are more amenable to EM than those from *S. cerevisiae*, and we provide views of complete, native kinetochores in ice. We identify the locations of microtubule-binding complexes and the inner kinetochore within the architecture of these particles and confirm the shape of these particles by real-time high-speed atomic force microscopy (HS-AFM). These particles are dynamic and much larger than any previously visualized kinetochore assemblies. This work lays the foundation for understanding how individual kinetochore complexes fit into a unified kinetochore architecture and how this architecture enables kinetochore functions.

## RESULTS

### Functional native kinetochores can be purified from *K. marxianus*

We set out to purify kinetochores from a thermophilic yeast to determine whether they are more stable for structural studies than those of *S. cerevisiae*. We first determined whether *K. marxianus* had homologs of the 40 established *S. cerevisiae* kinetochore proteins by identifying previously assigned kinetochore proteins in the NCBI *K. marxianus* database (NCBI:t-xid4911; Table S1; higher alignment scores and lower E values indicate better matches). For the 10 proteins that were not previously assigned, we identified homologs in *K. marxianus* by local alignment searches using either *S. cerevisiae* proteins or *Kluyveromyces lactis* proteins (Table S1). We detected all expected kinetochore proteins except Iml3 (CENP-L in humans), which is nonessential for vegetative growth but essential for meiosis.^48^

The strong similarity between the kinetochores in the two yeast species suggested that our previously described method to purify *S. cerevisiae* kinetochores via a one-step purification of the Dsn1 kinetochore protein might work in *K. marxianus*.^16^ We therefore tagged the *K. marxianus DSN1* gene at the endogenous locus with 6xHis and 3xM3DK epitopes (Dsn1-His-M3DK). Cells were treated with benomyl, which suppresses microtubule dynamics, to enrich for mitotic cells and to reduce cell-cycle stage variability. Kinetochores were captured from lysates using α-M3DK beads and then eluted with M3DK peptide. The elutions produced consistent banding patterns as visualized on a silver-stained gel (Figure 1B). We performed mass spectrometry to identify the co-purifying proteins and confirmed the presence of proteins from every kinetochore subcomplex except the nonessential Cnn1c (Figure 1C; Table S1). Kinetochore complexes were significantly enriched compared with purifications from negative control strains lacking the M3DK tag on Dsn1 (Figure S1A). Outer kinetochore proteins tended to be slightly overrepresented in terms of peptide-spectrum matches (PSMs), consistent with the observation that outer components are present at higher copy numbers.^49^ To further confirm that specific kinetochore proteins co-purified with Dsn1, we generated antibodies to representative members of the Dsn1 complex (Nnf1), the outer kinetochore Ndc80c (Spc24), and the inner kinetochore CCAN (Okp1) and performed immunoblotting on the purified particles. We detected these components, demonstrating that the purified particles spanned the inner to outer kinetochore (Figure 1D). To ensure that a stable kinetochore complex was purified, we fractionated the kinetochore eluate on a 20%–60% glycerol gradient and analyzed the fractions by immunoblotting. The gradient separated unbound kinetochore complexes from a small pool of assembled kinetochores (Figures S1B and S1C), demonstrating a population of stable and relatively complete kinetochores.

To test whether the *K. marxianus* native kinetochores could bind to microtubules under force, we used a previously established optical trapping assay that measures kinetochore-microtubule attachment strength.^16^ Kinetochores were conjugated to beads and introduced to microscope slide chambers with dynamic microtubules seeded on the coverslip surface. A single bead was trapped by a focused infrared laser and brought into proximity of a single microtubule tip to create a kinetochore-microtubule attachment. The interaction strength was measured by applying tension until the bead detached from the microtubule (rupture event), the maximum strength of the trap was reached, or the measurement was otherwise interrupted (escape event). *K. marxianus* kinetochores exhibited robust microtubule attachments with a similar median rupture force to kinetochores purified from *S. cerevisiae*—median rupture forces of 7.5 and 7.2 pN, respectively (Figure 1E). However, the distribution of events for *K. marxianus* appeared bimodal, with a high strength cluster that does not appear in *S. cerevisiae*. Despite the similarity of the median rupture forces, this distribution difference is significant by a log-rank test (*p* = 0.04), and its source is an area of future investigation. Together, these data show that *K. marxianus* kinetochores can maintain attachments at slightly higher forces than *S. cerevisiae*, confirming their functionality.

### Two classes of kinetochores are visible by EM

**Figure 2.**
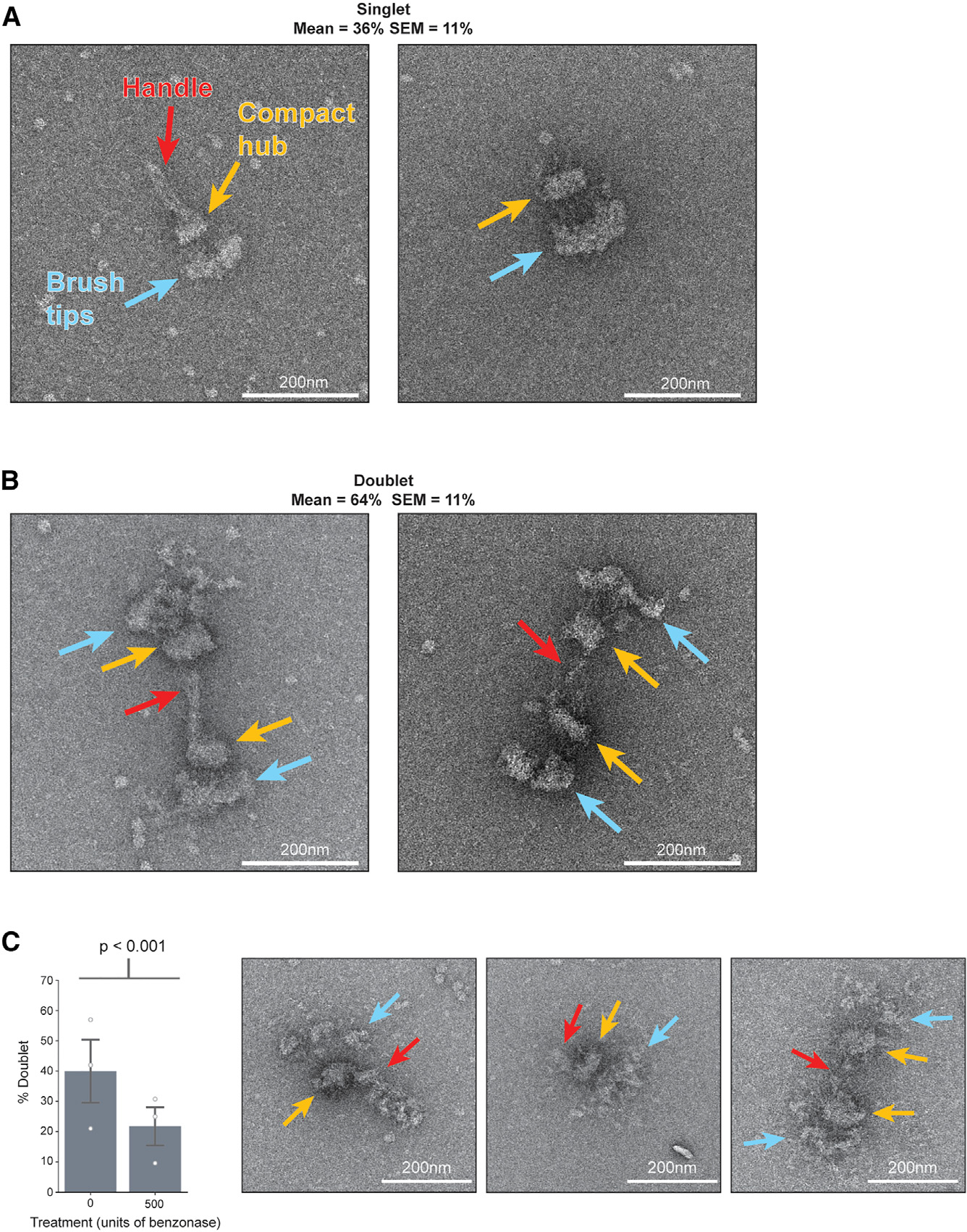
*K. marxianus* kinetochores have a paintbrush-like architecture and exist as singlets or doublets whose proportions are changed by nuclease treatment (A) Two representative negative stain electron micrographs of singlet kinetochores exhibiting three major regions: the brush tips (blue arrows), the compact hub (yellow arrows), and the handle (red arrow). (B) Two representative negative stain electron micrographs of doublet kinetochores. Arrows indicate the same major regions as (A). Quantification of the proportion of doublet and singlet kinetochores was done for 50 kinetochores across 5 biological replicates. The mean percentages of doublets and singlets were 64% and 36%, respectively. (C) Left: graph represents the percent of doublet kinetochores visualized by negative stain EM when compared with the total amount for mock-treated (0 units benzonase; *n* = 181 kinetochores over 3 biological replicates; mean = 40%; SEM = 10%) or nuclease-treated (500 units benzonase; *n* = 223 kinetochores over 3 biological replicates; mean = 22%; SEM = 6.3%) samples. White dots represent biological replicates and gray bars represent SEM. *p* = 3.4 × 10^−6^ by chi-squared test. Right: representative images of kinetochores treated with 500 units of benzonase. Arrows indicate the same features as (A). Scale bars, 200 nm. See also Data S2.

We next performed negative stain EM as a first step toward elucidating kinetochore architecture. Purified kinetochores were deposited on EM grids, stained with uranyl formate, and then imaged. Kinetochores appeared as large paintbrush-like structures with a flared “brush” end and a more compact hub, which often had a long, thin projection, akin to a paintbrush handle (Figure 2). A population of these kinetochores appeared as doublets, with two brushes connected by their handles (Figure 2B). The average proportion of doublets was higher than singlets (Figure 2; 64% doublets, 36% singlets; SEM = 11%; *n* = 50 kinetochores). We occasionally saw singlet kinetochores without a handle projection, which may be due to degradation or may represent an intermediate between singlets and doublets.

A key to interpreting the architecture of the kinetochore particles was to establish the polarity of inner and outer kinetochore regions. The branched architecture visible by negative stain appeared consistent with the hierarchical structure proposed for the kinetochore, with relatively few copies of inner kinetochore complexes building to many copies of outer kinetochore complexes.^42,50^ We therefore hypothesized that the wider brush end (Figure 2, blue arrows) consisted of the outer kinetochore-microtubule binders and the more tapered regions contained the inner kinetochore (Figure 2, orange arrows). The inner kinetochore could potentially contain the centromeric DNA. If this were true, nuclease treatment might degrade the compact hub or the handle. To test this, we performed a short, room temperature incubation with or without nuclease (benzonase). We then removed the benzonase with the final washes before eluting the kinetochores from beads. Although the compact hub remained largely intact, the proportion of doublets decreased significantly from 36% to 21% (*p* < 0.001; Figure 2C). The shift from doublets to singlets with nuclease treatment suggests that there is exposed nucleic acid in the handle of the kinetochores and that it is partially responsible for maintaining the doublet connection.

### Kinetochores interact with microtubules through the brush tips

**Figure 3.**
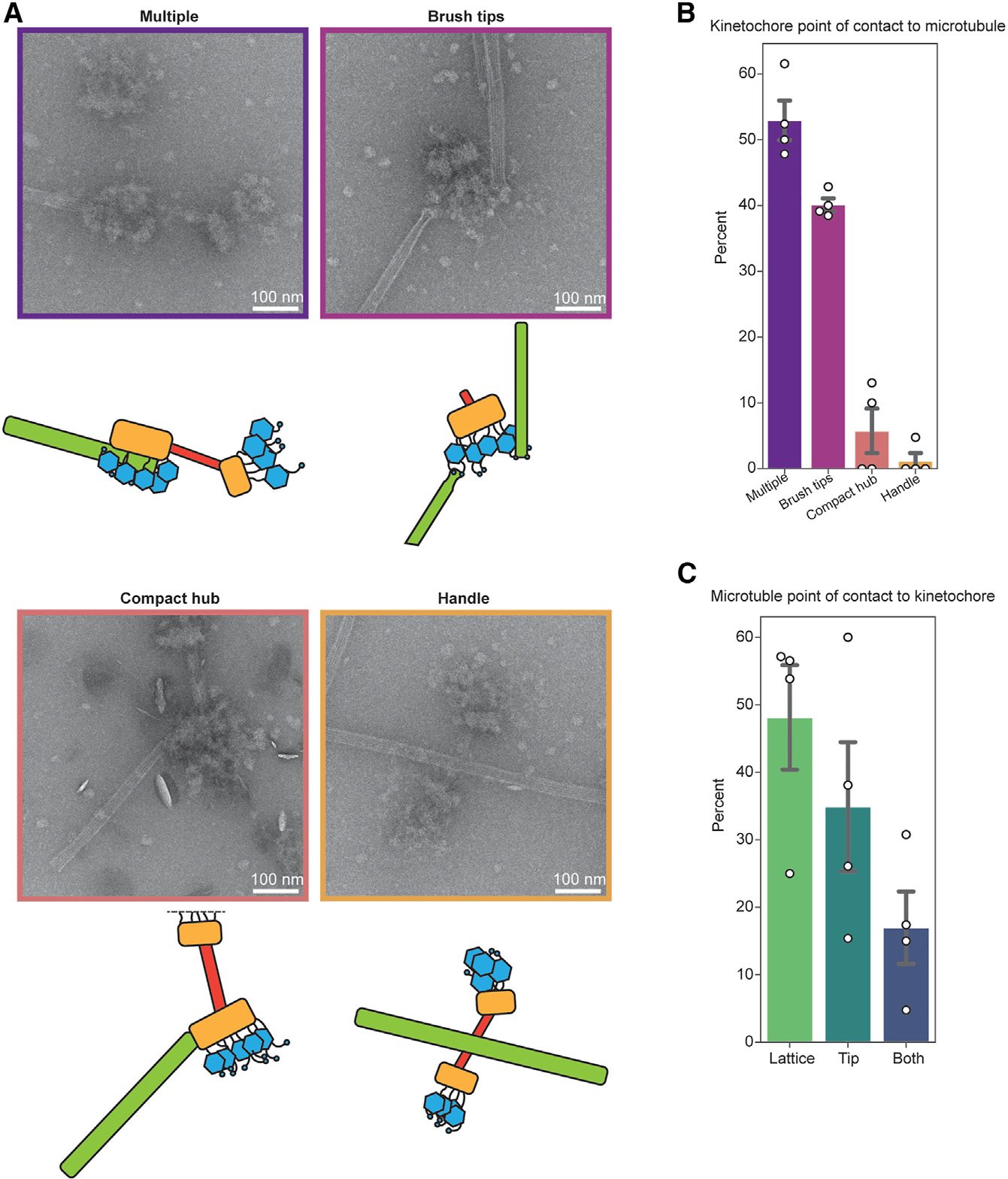
Kinetochores bind to the side and the ends of microtubules through the brush tips (A) Representative micrographs and corresponding cartoons showing multiple modes of interaction between kinetochores and taxol-stabilized microtubules. Images are representative of the categories in (B), and border colors correspond to the appropriate categories in (B). (B) Quantification of the percent of kinetochores seen interacting with the microtubule exclusively through the brush tips, handle, or compact hub. For (B) and (C) *n* = 91 kinetochores across 4 biological replicates. The multiple category includes microtubule-bound kinetochores, which appear to have multiple regions contacting the microtubule. White dots represent individual replicates. Gray bars represent the standard error of the mean. Mean percentages were as follows: multiple, 52.9%; brush tips, 40.1%; compact hub, 5.8%; handle, 1.2%. (C) Left: quantification of the percent of kinetochores interacting with the lattice, the tip, or both regions of microtubules. White dots represent individual replicates. Gray bars represent the standard error of the mean. Mean percentages were as follows: lattice, 48.1%; tips, 34.9%; both, 17.0%. Scale bars represent 100 nm. See also Figure S2 and Data S3.

Our discovery that the handle is sensitive to nuclease suggested that the brush tips contained the outer kinetochore (Figure 2, blue arrows). To test this, we incubated purified kinetochores with taxol-stabilized microtubules and imaged them by negative stain EM. We visualized interactions of the brush tips with the microtubules (Figure 3A). In cases where only a single region of the kinetochore contacted a microtubule, the brush tips were the most common point of contact (Figure 3B; 40% compared with 5.8% and 1.2% for the compact hub and handle, respectively). However, it was not possible to normalize these data to account for the differences in 3D surface area between the regions of the kinetochore because these data cannot be calculated from 2D images. In more rare views, either the compact hub or the handle could occasionally be seen exclusively contacting the microtubule (Figures 3A and 3B). We also asked whether the kinetochores had a preference between binding to the microtubule lattice or the tip by quantifying the position of the kinetochore on the microtubules and found there were no significant trends (Figures 3C and S2). Despite clear contact between the kinetochores and the microtubules, we were not able to detect the presence of Dam1c rings that were visualized in *S. cerevisiae* EM experiments.^43^ However, Dam1c components were present in the mass spectrometry data even though they did not oligomerize around the microtubules. Due to the dense clouds of proteins at the brush tips, it was also difficult to distinguish individual microtubule-binding sites on the kinetochores. Regardless, these data suggest that the brush contains the outer kinetochore complexes and that the purified kinetochores can bind to both the sides and ends of the microtubule.

### Ndc80 is located in the brush

**Figure 4.**
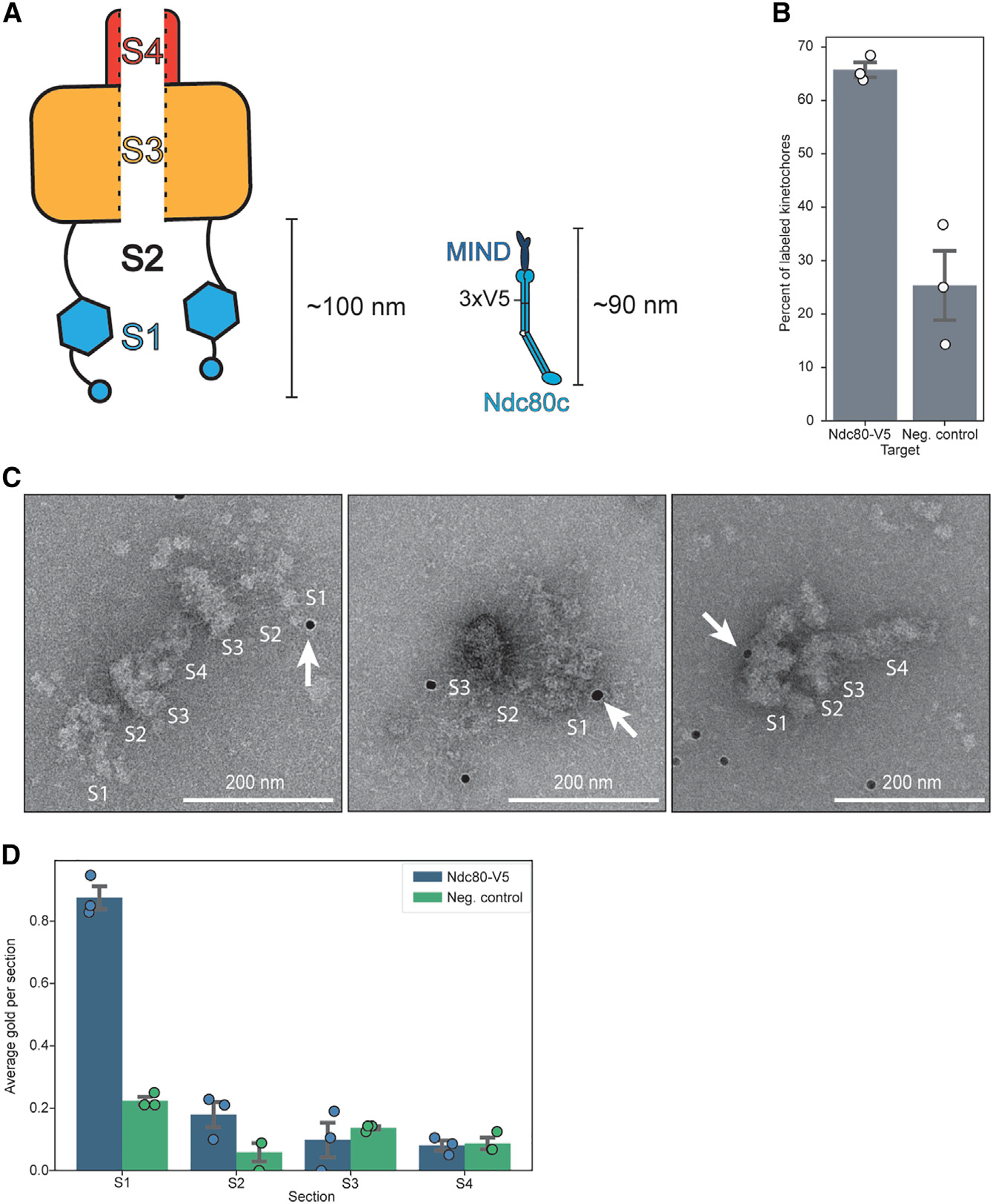
The major microtubule-binding complex is in the brush tips (A) A cartoon schematic depicting a kinetochore particle with the length of the suspected outer kinetochore region, along with a cartoon of the MIND/Ndc80c complex for comparison. The S1 region corresponds to the large density at the brush tips, S2 corresponds to the fibrils connecting the brush tips and the compact hub, S3 corresponds to the compact hub, and S4 corresponds to the handle-like projection. A 3xV5 label has been placed in its approximate location of the tag in the Ndc80-V5 strain (strain SBY21752). (B) A graph showing the percent of kinetochores with at least one associated α-V5 gold particle relative to the total number of kinetochores. Kinetochores were purified from a strain with Ndc80-V5 epitope-tagged (Ndc80-V5, SBY21752; mean = 66%; SD = 3.3%) or not tagged (neg. control, SBY18150; mean = 25%; SD = 11%), and gold labeling was performed. Both groups contain 3 biological replicates. Error bars represent the SEM, and white dots represent individual data points. (C) Representative micrographs from gold labeling experiments targeting Ndc80-V5 kinetochores. Kinetochores are broken into sections as in (A). White arrows indicate a gold label in contact with a kinetochore. Scale bars, 200 nm. (D) Quantification of the average amount of gold labels found in each section of the kinetochore as illustrated in (A) across 3 biological replicates. Individual data points are represented by dots, and error bars represent the standard error of the mean. Blue indicates the Ndc80-V5 sample, while green indicates the negative control that contains no V5 tag. See also Data S4.

We next sought to determine whether the major microtubule binder Ndc80c is in the brush region by gold labeling, as the brush was long enough to contain the MIND/Ndc80c rod (Figure 4A). We engineered a strain with a 3xV5 C-terminal tag on Ndc80p (Ndc80-V5) and then purified kinetochores from strains with or without the epitope-tagged Ndc80. To perform the labeling, we conjugated V5 antibody to 10 nanometer gold beads, incubated the beads with purified kinetochores, and then subsequently applied them to grids for negative staining. Ndc80-V5 kinetochores showed higher levels of gold labeling than those lacking the V5 tag (Figure 4B). We quantified the location of gold beads on visible kinetochores, and there was a significant enrichment of gold particles in the distal region of the brush tips (Figure 4D, region S1). These data suggest that Ndc80c is in the brush; however, the low level of gold labeling prevented us from identifying its location more specifically.

The negative stain data showed fibrils connecting the brush tips and compact hub (Figure 4A, region S2). These fibrils appear relatively rigid with an average length of roughly 54 nm. When added to the average length of the bulky brush tip density, roughly 42 nm, these two sections span nearly 100 nm (Figure 4A, regions S1 and S2). The Ndc80c is expected to be roughly 60 nm long, and when bound to the MIND complex, the two form a roughly 90 nm rod.^33,51–53^ The fibrils and brush tips are therefore consistent with the length and shape expected of MIND attached to Ndc80c. The significant density at the bush tips is likely due to partner proteins known to bind with or near Ndc80c, such as Dam1c; Stu2; the large, disordered tail of Spc105; and Mps1.^52,54–58^

### AFM reveals kinetochore dynamics

**Figure 5.**
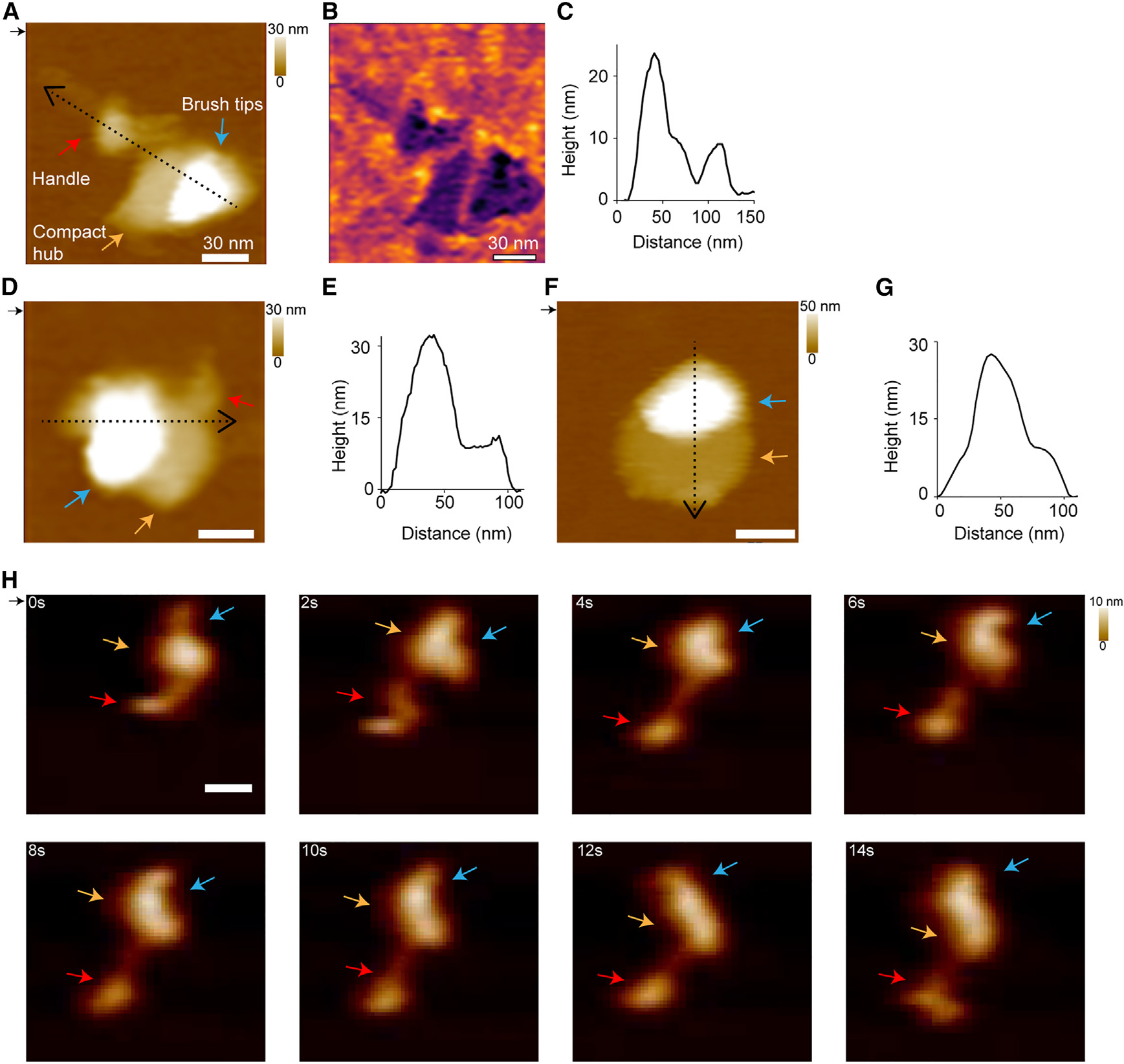
Atomic force microscopy confirms kinetochore shape and reveals dynamics (A) AFM height image of a kinetochore showing a paintbrush-like architecture with a long handle extending from a brush head. The red arrow indicates the brush handle, the orange arrow indicates the compact hub, and the blue arrow indicates the brush tips. (B) The phase image from (A). (C) The corresponding height profile taken along the dotted line in (A). (D) AFM height image of a kinetochore showing the long brush handle close to the brush head. (E) The corresponding height profile from the dotted line in (D). (F) Height image of a kinetochore with just the brush head. (G) The corresponding height profile from the dotted line in (F). (H) Successive AFM height images showing the dynamics of individual kinetochores with significant changes observed in the handle and the compact hub/brush tip regions over time (Videos S1 and S2). The direction of the scan is indicated by the small black arrow at the top left of the images. The scanning rate is 2 min/frame in (A), (B), (D), and (F) and ~1 s/frame in (H) and (I) with 256 × 256 pixels. The x-y scale bar is 30 nm. The z scale is 0 to 30 nm (dark to light brown) in (A) and (D), 0 to 50 nm in (F), and 0 to 10 nm in (H). The AFM image is colored according to height from the surface. See also Figure S3 and Videos S1 and S2.

To confirm the architecture using a different method as well as to examine kinetochore dynamics, we employed AFM. AFM can provide information on dynamics and flexibility within individual kinetochore molecules. Kinetochore particles were deposited on a mica surface and imaged. We first examined the surface topography of the individual kinetochore particles by imaging at a relatively slow scan rate (~5 Hz). We classified the observed structures into three categories by visual examination. The first category included particles with extended architecture. Consistent with the negative stain images, we observed a paintbrush-like architecture with a long handle extending from a brush head (Figures 5A–5C; 50% [*n* = 68]). The second category consisted of more compact particles where the handle appears to be close to the brush head (Figures 5D and 5E; 21% [*n* = 29]). The third category had particles where the handle was not visible (Figures 5F and 5G; 29% [*n* = 40]). In addition, we occasionally observed doublets like those observed by electron microscopy (Figure S3). The different extended and closed structures observed by AFM could arise from differences in the orientation of the particles on the mica surface. Alternatively, they may represent inherent dynamics and flexibility of the kinetochore. To distinguish between these possibilities, we performed HS-AFM by imaging the same sample at a higher scan rate (100 Hz) to better visualize the dynamics of the particles. The time-lapse data revealed conformational dynamics in the kinetochore particles, with significant changes in the observed extension of the handle regions over time (Figure 5H; Videos S1 and S2). This is consistent with extended and compacted states observed by low-speed AFM imaging (Figures 5A–5G) and with the flexibility seen in negative stain EM. Dynamics were also observed in the brush head segment (Figure 5H; Videos S1 and S2).

### Kinetochores are visible by cryo-ET

**Figure 6.**
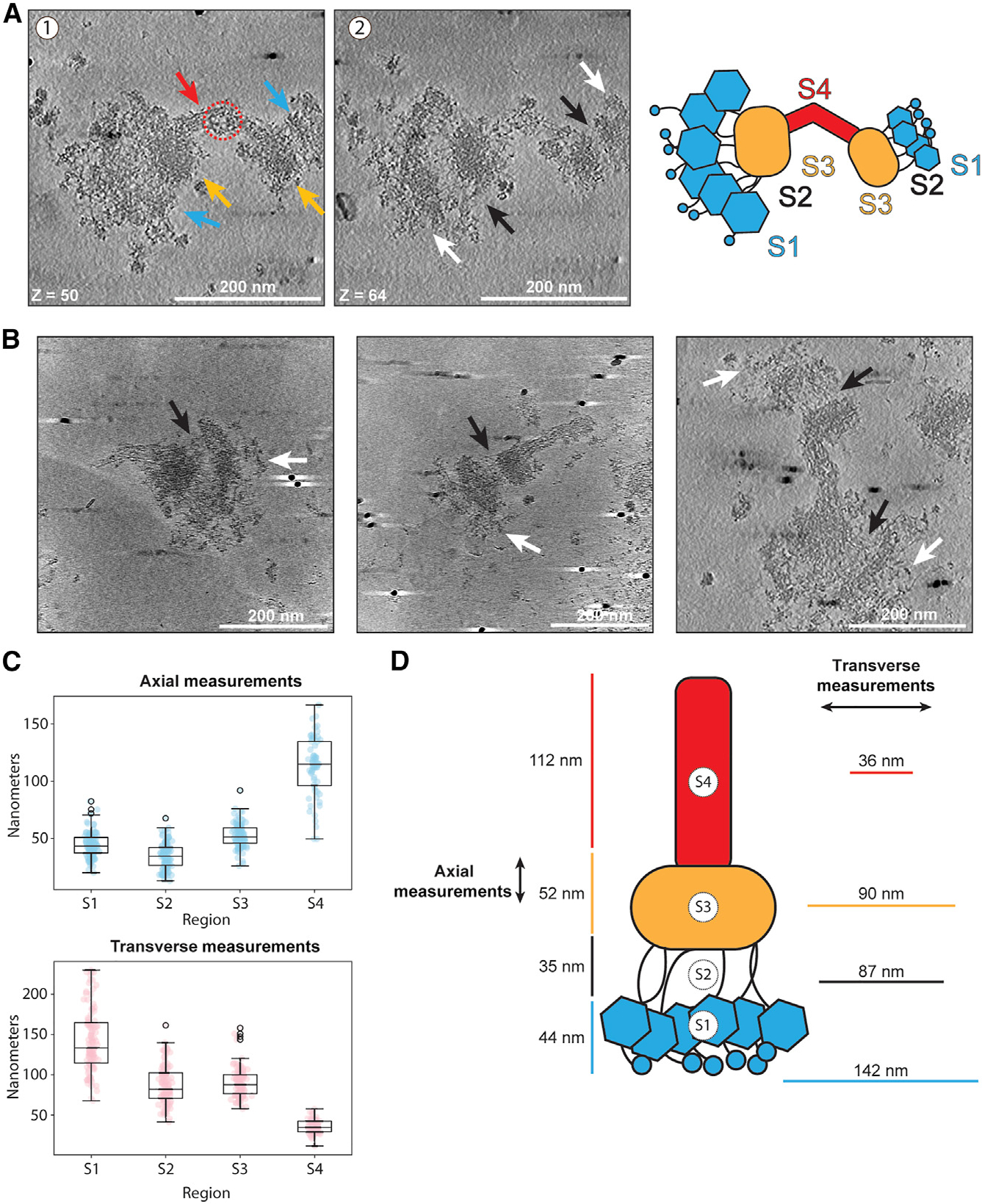
Cryoelectron tomography data reveal the major regions of kinetochore architecture (A) Two slices of a representative tomogram of a doublet kinetochore. (1) Arrows indicate the presence of the same major regions as seen by negative stain—the brush tips (blue), the compact hub (yellow), and the handle (red). The red dashed circle indicates the donut-like gap that could occasionally be seen in the handle. (2) Features that were not apparent in negative stain became more visible in ice. White arrows indicate brush tip extensions; black arrows indicate flexible fibrils connecting the brush tips to the compact hub. Scale bars are 200 nm, and Z slice numbers are indicated at the bottom left of each image. Each Z step is 1.056 nm. Far right: schematic of kinetochore sections. (B) Example slices from tomograms of 3 different kinetochores. Black arrows indicate flexible fibrils, and white arrons indicate brush tip extensions. (C) Distance measurements of 101 kinetochores at the regions specified in the cartoon in (A) were taken axially (top, blue) or transversely (bottom, pink) and plotted as boxplots. Colored dots indicate individual data points. Boxes indicate the interquartile range (25%–75%) with the median indicated by a black line inside the rectangle. Whiskers extend from the box to 1.5× the interquartile range, and outliers are indicated by white circles with black edges. (D) Average kinetochore distance for each section. Axial measurements are to the left of the cartoon, and transverse measurements are to the right. See also Figures S4 and S5, Videos S3 and S4, and Data S5.

Given the success in visualizing the kinetochores via negative stain, we sought higher resolution information. However, although the *K. marxianus* kinetochores were more abundant on grids than previous attempts with *S. cerevisiae*,^43^ it was still rare to image a negative stain grid with more than 100 total identifiable kinetochores. Due to the extremely low number of visible particles on grids, single particle cryo-EM was not an option. We therefore turned to cryoelectron tomography (cryo-ET) to maximize the information attained from each particle imaged. To increase the kinetochore concentration on grids, we employed magnetic isolation and concentration-cryo-EM (MagIC-cryo-EM), which uses magnetic nanobeads to capture and pull kinetochores to the grid surface using a strong magnet.^59^ For unknown reasons, the kinetochores did not bind well to the magnetic nanobeads. However, the presence of the nanobeads improved the quality of the ice, leading to more visible kinetochores. We were able to collect over 100 tomograms of kinetochores that maintained the key features visible by negative stain and occasionally showed an additional layer of density distal to the bulk of the brush tips (Figures 6A, 6B, and S4; Videos S3 and S4). Although we made repeated attempts at sub-tomogram averaging using various strategies, they did not provide sufficient EM density maps to build structural models. This is likely due to kinetochore flexibility and heterogeneity combined with difficulty in maintaining kinetochore integrity in thin ice and a strongly preferred orientation, which limited the potential particle views. Despite the inability to perform averaging, these tomograms provided improved views of several regions of the kinetochore (Figures 6A and 6B). The linkages between the brush tips and the compact region appear as long and relatively rigid fibrils that are easier to distinguish compared with the negative stain images. They have a consistent length of about 35 nm. When the fibril length is added to the length of the bulky brush tip density (45 nm), the combined length is ~80 nm. This is slightly smaller than the ~100 nm measured for this region by negative stain but is still consistent with a roughly 90 nm rod like MIND/Ndc80c complex. The additional length in negative stain may be due to the stain flattening the proteins. The handle also showed more features than were visible in negative stain, revealing fibrous strands running parallel to the length of the handle. Occasionally, a gap or donut-like structure appeared at the center of the handle (Figure 6A, red arrow). The cryo-ET images also made it apparent that the handle does not protrude exactly from the center of the compact region but rather from the outside edge. Quantifying the sizes of these kinetochores confirmed that they are highly flexible, apparent from the spread of measured distances, particularly along the transverse axes of the brush tips and fibrils (Figure 6C, regions S1 and S2). They are also larger than those previously measured from *S. cerevisiae* with an axial length of roughly 243 nm (Figures 6C and 6D).^43,60^ Interestingly, omitting the handle from these measurements brings the average axial length to roughly 131 nm, close to that reported in *S. cerevisiae*.^43,60^ Together, these data are consistent with the predicted size and shape of kinetochores based on previous structural work and suggest a general conservation of kinetochore structure.

## DISCUSSION

**Figure 7.**
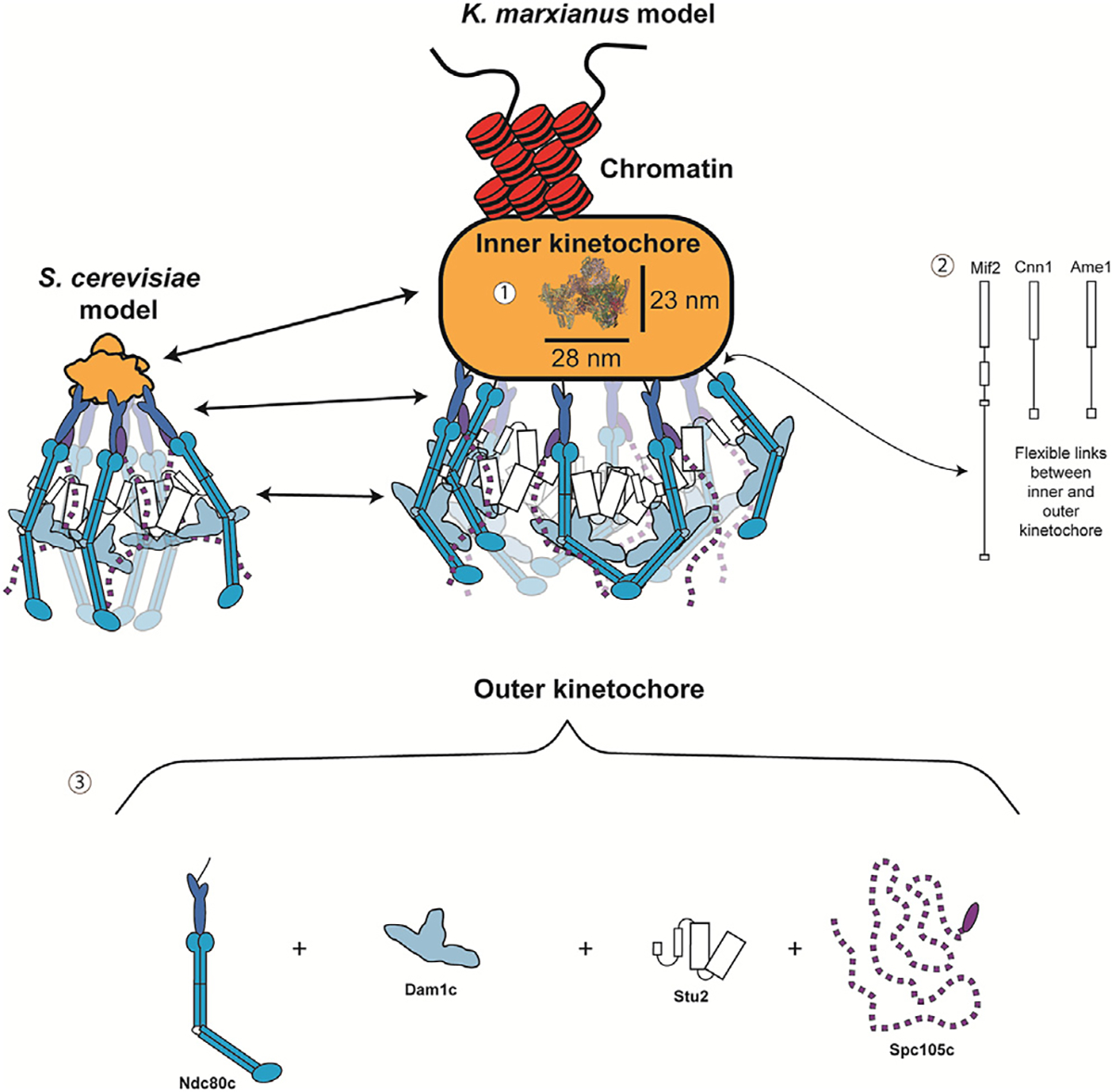
Key insights into global kinetochore architecture A roughly to scale comparison of the size and overall architecture of the existing *S. cerevisiae* kinetochore model (left) compared with brush-like particles of this work (right). Several features are highlighted by these kinetochores: (1) the inner kinetochore is buried within a dense mass large enough that published CCAN structures that include 2 CCANs and a nucleosome (PDB: 8OW1) can fit within its dimensions. There is also an associated handle structure that contains exposed nucleic acid. (2) The connections between the inner and outer kinetochore are made by flexible regions of Mif2, Ame1, and Cnn1, which allow for the high flexibility of the outer kinetochore. (3) The major microtubule binder, Ndc80c, is in the brush tips. The rigid fibrils connecting the central hub and the brush tips are likely MIND and a portion of Ndc80c, and Ndc80c’s interaction with various large and often flexible binding partners creates large clouds of proteins capable of binding microtubules and regulating those interactions. See also Figure S5.

Here we report the purification of relatively complete native kinetochore particles from the thermophile *K. marxianus* that retain microtubule-binding ability. Though we did not detect Cnn1c in our purifications, this complex has also been difficult to detect by mass spectrometry in *S. cerevisiae* kinetochore purifications, and we lacked an antibody to probe for it by immunoblot, so it is unclear whether it is present. The *K. marxianus* particles were more stable than those purified from *S. cerevisiae*, allowing us to collect views of kinetochore particles by cryo-ET and AFM. The kinetochores had a paintbrush-like overall architecture, with many brush tips tapering into a compact hub and handle-like structure (Figure 7). In addition, the brush tips and handle were flexible when analyzed by AFM. Using microtubule-binding assays, we determined that the outer kinetochore region resides in the brushes, which have a size and shape consistent with Ndc80c and its associated proteins. We also identified nucleic acid within the handle through nuclease treatment, confirming it as adjacent to the inner kinetochore. This established polarity within these kinetochore images and suggested the flexibility of the handle may be due to chromatin.

One striking feature of the *K. marxianus* kinetochores is their size. The average axial length of a minimum kinetochore unit is ~131 nm, omitting the handle as kinetochores were occasionally seen without it. The average axial length of the handle alone is 112 nm, significantly increasing the overall size. However, the significant variability in the handle measurements and the dynamics seen by AFM suggest that the handle is very flexible and capable of undergoing compaction. Although the length of the *K. marxianus* kinetochore without the handle is close to isolated *S. cerevisiae* kinetochores (126 nm),^43^ the EM measurements are larger than those attained by *in vivo* fluorescence work in *S. cerevisiae* that described a roughly 70 nm metaphase kinetochore and a 50 nm kinetochore in anaphase.^60^ Some of this discrepancy may be due to EM methods. For example, negative staining can flatten complex protein assemblies, leading to some distortion or elongation,^61^ and cryo-ET sample preparation may compress the kinetochore, causing it to spread out. Despite these size differences, the MIND/Ndc80c proteins appear to form a 90 nm rod like structure, which is consistent with the shape and length of our proposed outer kinetochore. In the future, resolving these questions will require generating sufficient electron density maps to build atomic models of the subcomplexes within the structure.

The native kinetochores we visualized exhibit a more complicated architecture than previously published structures. The average axial and transverse lengths of the compact hub are roughly 52 and 90 nm, respectively. This is large enough to easily contain the largest published assembly of the yeast inner kinetochore, containing two CCANs, the centromeric nucleosome, and a core Cbf3 complex.^62^ However, the high flexibility and density of proteins within the kinetochores made it impossible to assign proteins or identify a CCAN-like structure. There are several possible reasons for this difficulty. First, native kinetochores retain much more protein than recombinant subcomplexes. Second, chromatin compaction could create a dense environment that obscures the CCAN. Consistent with this, chromatin has been shown to tightly compact *in vitro* at magnesium chloride concentrations similar to those present in the purification buffer.^63^ We were also unable to discern the KMN network in the tomograms. However, additional large, flexible proteins bind to various members of the KMN and may make up the additional brush tip density.^52,53,55,56^

Another feature of kinetochores we identified is high flexibility of the connections between the inner and outer kinetochore. In yeast, these critical links occur through the essential proteins Ame1 and Mif2.^30,31,50,64,65^ The connection between these proteins and the outer kinetochore occurs via domains predicted to be unstructured (Figures 7 and S5). This is apparent in the cryo-ET through the wide variety of configurations detected, but particularly in the spread of the transverse length measurements for the outer kinetochore (Figure 5C, region S1) and the axial length measurements of the handle (Figure 6C, region S4). Dynamics in both regions were directly observed by HS-AFM. The flexibility of the outer kinetochore may allow the microtubule-binding elements to spread, increasing the effective surface area that can contact the microtubule to facilitate capture. The flexibility may also assist the kinetochore in maintaining contact with the microtubule in sub-optimal orientations. The handle also appears to extend as seen by AFM, possibly due to the unraveling of chromatin. Further exploration as to the nature and composition of the handle will be needed to better understand these dynamics.

An unexpected finding from this work was the presence of doublet and singlet kinetochores. Because nuclease treatment reduces the proportion of doublets and singlet kinetochores can occasionally be seen without visible handles, we propose that the functional kinetochore unit contains the brush tips to the compact hub and the handle links kinetochore units through their inner regions via nucleic acid. The average sizes of the individual kinetochore units within doublets are consistent with the average size of singlet kinetochores, but they are not identical in size. Furthermore, both kinetochore units in doublets can independently interact with microtubules, suggesting each is functional. The handle connecting doublets also shows interesting features. Its width is remarkably consistent when compared with the widths of the rest of the kinetochore, and its dimensions are not far from what might be expected from a 30 nm chromatin fiber that forms *in vitro* at salt concentrations similar to our buffers.^63^ In some cases, the handles also show a ring-like gap, potentially indicating some kind of linkage or hinge point. We propose three hypotheses for the functional role of doublets. First, *K. marxianus* may have small regional centromeres that bind to multiple microtubules, and a doublet would bind to two microtubules. Another possibility is that each doublet binds to one microtubule. While *K. marxianus* and *S. cerevisiae* centromeres both contain three centromere-defining elements (CDEI, CDEII, and CDEIII), the length of CDEII in *K. marxianus* is nearly double that of *S. cerevisiae*^66^ and may represent an important feature for building doublet kinetochores. A third possibility is that the doublets are sister kinetochores that were not broken apart during the purification process. However, proteins that link sister kinetochores such as cohesin were not readily detectable in our mass spectrometry data. Distinguishing these ideas will require determining the number of microtubule-binding sites per centromere *in vivo*.

In the work presented here, we utilized a thermophilic yeast with conserved kinetochore proteins to purify native kinetochores that were amenable to structural biology. These kinetochores bear a striking similarity to the tri-laminar structure reported in the earliest EM images of mammalian kinetochores,^67–69^ with the inner plaque, translucent layer, and outer plaque mirroring the central hub, fibrils, and brush tips, respectively. The images also highlight the size and complexity of the kinetochore as it exists in its native environment and demonstrate that recombinant subassemblies are much simpler than the native structures. We were able to capture clear views of kinetochore interactions with microtubules, which will serve as the basis for understanding how many subcomplexes engage the microtubule tip, the details of how they do so, and how this process is regulated. Together, this work serves as the foundation for understanding the architecture of complete kinetochores and how that architecture allows for its essential functions.

## STAR★METHODS

### RESOURCE AVAILABILITY

#### Lead contact

Further information and requests for resources should be directed to and will be fulfilled by the lead contact, Sue Biggins (sbiggins@fredhutch.org).

#### Materials availability

Yeast strains and reagents generated by this study are available upon request.

#### Data and code availability

- Mass spectrometry data generated in this study is available through Mass Spectrometry Interactive Virtual Environment (MassIVE, University of California San Diego). The published article includes all other datasets generated in this study as Data Supplements Data S1, S2, S3, S4, and S5.
- This paper does not report original code.
- Any additional information required to reanalyze the data reported in this paper is available from the lead contact upon request.

### EXPERIMENTAL MODEL AND STUDY PARTICIPANT DETAILS

*Saccharomyces cerevisiae* strains used in this study are derived from W303. All *Kluyveromyces marxianus* strains are derived from NRRL Y-8281. Yeast were grown initially at 30 °C and shifted to 23 °C during benomyl treatment unless otherwise indicated. Yeast were cultured in standard yeast extract peptone dextrose (YPD) unless otherwise indicated. Strains used in this study are listed in the key resources table.

### METHOD DETAILS

#### Strain construction

The *Saccharomyces cerevisiae* strain used in this study is SBY8253 (*DSN1–6xHis-3xM3DK:URA3*) and was derived from the W303 background and was previously described.^16^ The *Kluyveromyces marxianus* strains used in this study are SBY18150 (*DSN1–6xHis-3xM3DK:KanMX*) and SBY21752 (*NDC80–6xHis-3xV5:NatMX*, *DSN1–6xHis-3xM3DK:KanMX*) and were derived from SBY17411 (NRRL Y-8281, USDA ARS culture collection). All strains were tagged at the endogenous locus. Briefly, DNA fragments of either 500 or 1000 bases immediately upstream and downstream of the desired integration site were generated from genomic DNA. A backbone plasmid was selected based on the desired tags, and the fragments amplified from genomic DNA were inserted into the backbone plasmid via Gibson assembly such that each plasmid contained a restriction site, followed by 500 to 1000 base pairs of upstream homology, followed by the desired tags and markers, followed by 500 to 1000 base pairs of downstream homology, followed by another restriction site. Plasmids were then digested and transformed into the desired strain for integration by homologous recombination. Successful integration was confirmed by PCR and immunoblotting. The plasmids (pSB prefix) and yeast strains (SBY prefix) used are as follows: SBY18150 contains plasmid pSB2951 generated with primers SB5736, SB5737, SB5738, SB5739, transformed into SBY17411; SBY21752 contains plasmid pSB3420 generated with primers SB7818, SB7819, SB7820, SB7821, SB7822, SB7823, transformed into SBY18150. All tagged strains grow similarly to the parent strain.

#### Yeast growth and kinetochore purification

All yeast growth was performed as described previously.^16^ Briefly, yeast were grown in YPD (1% yeast extract, 2% peptone, 2% D-glucose). SBY18150 cultures were grown in the presence of 200 μg/ml G418. SBY21752 cultures were seeded from colonies grown on plates containing 100 μg/ml Nourseothricin Sulfate and were grown in the presence of 200 μg/ml G418 in liquid cultures as selection markers. Large cultures were grown on shakers (220 rpm) at 22 °C or 30 °C for *S. cerevisiae* and *K. marxianus*, respectively. Cultures were treated with benomyl at a final concentration of 30 μg/ml (1:1 addition of 60 μg/ml benomyl YEP media) for 2 hours at 23 °C and then harvested by centrifugation for 10 minutes at 5000×g at 4 °C. Kinetochores were purified as previously described.^16^ Briefly, the endogenous *DSN1* kinetochore gene was C-terminally tagged with 6xHis and 3xM3DK. Harvested yeast were resuspended in Buffer H (25 mM HEPES pH 8.0, 150 mM KCl, 2 mM MgCl2, 0.1 mM EDTA pH 8.0, 0.1% NP-40, 15% glycerol) supplemented with protease inhibitors, phosphatase inhibitors, and 2 mM DTT. After resuspension and re-spinning, yeast pellets were frozen in liquid nitrogen and lysed using a Freezer Mill (SPEX, Metuchen NJ). Lysate was clarified via ultracentrifugation at 24,000 RPM (98,000 × g) for 90 minutes and the protein layer was extracted with a syringe. This extract was incubated with magnetic α-M3DK antibody conjugated Dynabeads (Invitrogen, Waltham MA) for 90 minutes at 4 °C with rotation. For optical trapping, immunoblotting, and mass spectrometry, the Dynabeads were washed with 10x bead volume of Buffer H 5 times (the last 3 washes omitting DTT and phosphatase inhibitors). For optical trapping and immunoblots, kinetochores were eluted with 0.5 mg/ml 3xM3DK peptide in Buffer H lacking DTT and phosphatase inhibitors. For mass spectrometry, kinetochores were eluted from Dynabeads with 0.2% RapiGest (Waters Corporation, Milford MA) in 50 mM HEPES pH 8.0. For negative stain electron microscopy and cryo-electron tomography, kinetochores were washed with 10x bead volume of Buffer H 4 times (the last 2 washes omitting DTT and phosphatase inhibitors), followed by one wash in Buffer H-EM (25 mM HEPES pH 8.0, 150 mM KCl, 2 mM MgCl2, 0.1 mM EDTA pH 8.0) and elution with 0.5 mg/ml 3xM3DK (Genscript, Piscataway NJ) peptide in 1/3 the total volume of Dynabeads for negative stain experiments and ½ the bead volume for tomography. For all experiments, the total protein concentration was determined by NanoDrop measurement and purity by silver stain gel analysis.

#### Immunoblot and silver stain analyses

For immunoblot analysis, cell lysates were prepared as described above. Protein samples were separated using pre-cast 4–12% Bis Tris Protein Gels (Thermo-Fisher Scientific, Waltham MA) for sodium dodecyl sulfate-polyacrylamide gel electrophoresis (SDS-PAGE) in MOPS buffer pH 7.0 (20 mM MOPS, 5 mM sodium acetate, 1 mM EDTA). For immunoblotting, a 0.45 μm nitrocellulose membrane (BioRad, Hercules CA) was used to transfer proteins from polyacrylamide gels. The antibodies used for immunoblotting against Spc24, Nnf1 and Okp1 were custom generated by Genscript (Piscataway, NJ) against recombinant proteins that were expressed and purified from *Escherichia coli* and then injected into rabbits (Okp1: residues 190–440; Nnf1: full length protein; Spc24: full length protein). Genscript affinity purified the antibodies using the recombinant proteins and the resulting antibodies were used at the following dilutions: α-Spc24 used at 1:5,000; α-Nnf1 used at 1:2000; α-Okp1 used at 1:2000. Genscript services were also used to generate an M3DK antibody (Light chain variable region sequence: DVLMTQIPLSLPVSLGDQASISCRSSQSIVHRNGNTYLEWYLLKPGQSPKLLIYKVSNRFSGVPDRFSGSGSGTDFTLKISRVEAEDLGVYYCFQGSHVPYTFGGGTKLEIR, Mouse Ig kappa; heavy chain variable region sequence: QVQLQQSAAELARPGASVKMSCKASGYSFTTY TIHWVKQRPGQGLEWIGYINPSS GYAAYNQNFKDETTLTADPSSSTAYMELNSLTSEDSAVYYCAREKFYGYDYW GQGATLTVSS, mouse IgG2a) which was used at 1:10,000. The secondary antibodies used were a sheep α-mouse antibody conjugated to horseradish peroxidase (HRP) (GE Life sciences, Marlborough MA) at a 1:10,000 dilution or a donkey α-rabbit antibody conjugated to HRP (GE Life sciences, Marlborough MA) at a 1:10,000 dilution. Antibodies were detected using the Super Signal West Dura Chemiluminescent Substrate (Thermo-Fisher Scientific, Waltham MA). For analysis by silver stain, the gels were stained with Silver Quest Staining Kit according to manufacturer’s instructions (Invitrogen, Waltham MA).

#### Glycerol gradient

Glycerol gradients were prepared directly in thick-walled centrifugation tubes compatible with a SW55Ti rotor (Beckman Coulter, Brea CA). Gradients were made by stacking 400μl layers of 60%, 40%, and 20% glycerol in buffer H lacking NP-40 and including protease inhibitors (see yeast growth and kinetochore purification) and stored at 4 °C for 1 hour. Kinetochore elutions were loaded gently on top of the gradient at a volume between 100–300μl. Gradients were spun at 30,000 rpm for 16 hours at 4 °C. After spinning, gradients were fractionated manually into 100 μl fractions starting from the top (low density end) of the gradient. Fractions were then prepared for analysis by immunoblot or silver stain (see immunoblot and silver stain analysis).

#### Homology searches

To identify potential homologs of *S. cerevisiae* proteins in *K. marxianus* we performed a homology search. We used the *S. cerevisiae* kinetochore protein sequences available for the W303 background in the Saccharomyces Genome Database (SGD, Stanford University) and searched against known *K. marxianus* proteins (NCBI TaxonID:4911) using the Basic Local Alignment Search Tool for proteins (BLASTp, National Institutes of Health). In cases where no homolog was found by this method, protein sequences from *Kluyveromyces lactis* were used in place of *S. cerevisiae*.

#### Optical trapping

Optical trapping rupture force assays were performed as previously described.^16^ Streptavidin coated 440 nm polystyrene beads (Spherotech, Lake Forest IL) were functionalized with biotinylated α-penta-His antibody (Qiagen, Hilden Germany or R&D Systems, Minneapolis MN) and stored in BRB80 containing 8 mg/ml BSA and 1 mM DTT at 4 °C with continuous rotation. Beads were decorated with purified kinetochores (via Dsn1–6His-3M3DK) in a total volume of 20 μl incubation buffer (BRB80 containing 1.5 mg/mL κ-casein). To ensure sparse decoration of the beads and reduce the likelihood of multiple kinetochore-microtubule interactions being measured simultaneously, we empirically determined kinetochore concentrations such that roughly 1 in 10 beads exhibited microtubule binding activity during the assay. Dynamic microtubule extensions were grown from coverslip-anchored GMPCPP-stabilized microtubule seeds in a microtubule growth buffer consisting of BRB80, 1 mM GTP, 250 μg/ml glucose oxidase, 25 mM glucose, 30 μg/mL catalase, 1 mM DTT, 1.4–1.5 mg/mL purified bovine brain tubulin and 1 mg/mL κ-casein. Assays were performed at 23 °C. Rupture force experiments were performed as in Akiyoshi et al.^16^ Briefly, an optical trap was used to apply a force of ~1–2 pN in the direction of microtubule assembly. Once beads were observed to track with microtubule growth for roughly 30 seconds (to ensure end-on attachment), the applied force was increased at a constant rate of 0.25 pN/s until bead detachment. Records of bead position over time were generated and analyzed using custom software (LabVIEW:SCR_014325 and Igor Pro:SCR_014325, respectively) and used to determine the rupture force, which was marked as the maximum force sustained by the attachment during each event.

#### Negative stain electron microscopy

3–5 μl of purified kinetochores were taken directly from elutions and deposited on glow discharged (Pelco easiGlow, Ted Pella, Redding CA) 400 mesh electron microscopy grids (01754-F F/C, Ted Pella, Redding CA) for 1 minute. Grids were then washed twice with water and once with 0.75% uranyl formate before staining for 45 seconds with 0.75% uranyl formate and drying overnight. Grids were imaged using a Talos L120C 120kV transmission electron microscope with a 4k × 4k Ceta 16m CMOS camera (ThermoFisher Scientific, Waltham MA) at a magnification of 36,000x and a pixel size of 4.11 angstroms at a nominal defocus of 2 μm under focus.

#### Microtubule binding experiments

##### For microtubule binding experiments, microtubules were prepared as follows: purified bovine tubulin was incubated at a concentration of 2 μg/μl in BRB80 with 6 mM magnesium chloride, 1 mM GTP, and 3.8% DMSO. The tubulin was allowed to polymerize for 30 minutes at 37 °C and then 0.01 mM taxol in BRB80 was added at a volume equal to half that of the polymerization mixture (i.e. 100 μl for 200 μl of mixture) and mixed with wide bore pipette tips. The resulting mixture was then spun at 58,000 rpm for 10 minutes at 37 °C and the supernatant discarded. The pellet was resuspended with 0.01 mM paclitaxel in BRB80 at a volume equal to that of the original polymerization mixture. These microtubules were mixed 1:20 in eluted kinetochores and incubated for ~15 minutes at room temperature before applying to grids for negative staining.

#### Gold labeling experiments

α-V5 antibody was conjugated to 10 nm gold particles (Abcam, Cambridge UK) following the product recommended protocol. Kinetochores were purified as specified above for negative stain. Antibody conjugated gold particles were incubated with kinetochores at a ratio of 1:100 for 30 minutes at room temperature with rotation. This mixture was used to prepare grids as described above and imaged with the same parameters. Gold labeling was quantified by hand. A particle was considered a kinetochore if it discernable structure corresponding to at least regions S1, S2, and S3 as described in Figure 4A and was at least 100 nm long and 50 nm wide. A gold particle was determined to be in contact with a kinetochore if it was overlapping or within 10 nm of visible kinetochore density.

#### Nuclease digestion experiments

Kinetochores were prepared similarly to previously stated methods (yeast growth and kinetochore purification) apart from a benzonase treatment before the final two washes. Dynabeads were used to bind kinetochores from yeast lysate as described above. To keep the kinetochore concentration consistent across replicates, the lysate was diluted such that 50 μl of beads were used for every purification. Kinetochore-decorated beads were washed with 1 ml of Buffer H 4 times (the last 2 washes omitting DTT and phosphatase inhibitors). While on beads, the kinetochores were incubated with or without 500 units of benzonase (Millipore Sigma, Burlington MA) per milliliter of reaction volume. This mixture was rotated at room temperature for 15 minutes before washing the beads twice with buffer H-EM and elution in ½ the bead volume with 0.5 mg/ml M3DK peptide. Grids were prepared as described above. Quantification of the proportion of doublets was performed by hand.

#### CryoEM grid preparation

Kinetochore purification was performed as described above. Purified kinetochores were incubated with biotinylated α-His antibody (R&D Systems, Minneapolis MN) at an antibody concentration of 0.025 ng/μl for 1 hour at 4 °C with rotation. Samples were then mildly crosslinked using a final concentration of 0.01% glutaraldehyde for 15 minutes on ice and quenched with a final concentration of 50 mM tris pH 7.4 buffer for 15 minutes. The crosslinked kinetochore/antibody sample was incubated with 50 nm magnetic nanobeads (CD Bioparticles, Shirley NY) which had been conjugated to a 60 nm single α-helix spacer protein terminating in a SPY-tag, which had in turn been conjugated to a SPY catcher/avidin. The nanobeads were incubated with the kinetochores at a final concentration of 1 ug/ml. This mixture was incubated for 90 minutes at 4 °C, at which point it was spun at 12,000×g for 15 minutes at 4 °C to pellet. To minimize the loss of kinetochores that may not have been attached to beads, 2/3 of the supernatant volume was removed and the remaining 1/3 was used to resuspend the pelleted beads (as opposed to removing all the supernatant and resuspending in fresh buffer). The resuspended nanobeads were sonicated for 10 minutes in a water bath sonicator (Emerson, St. Louis MO) at 4 °C. 2.5 μl of sample was applied to 300 mesh Quantifoil R 2/1 copper grids (Electron Microscopy Sciences, Hatfield PA) that had been previously glow discharged (15 mA, 30 seconds; PELCO easiGlow, CA). Sample was incubated for 30 seconds at room temperature before manually wicking with a filter paper and re-applying. The grid was then held over a strong magnet for 30 seconds before being placed into a Vitribot Mark IV and blotted with the following settings: blot time:3; blot force: 7; wait time: 0; temperature: 22°C; humidity: 100%. 2 filter papers were additionally used per blotting surface in the Vitribot.

#### Cryo-electron tomography data collection and image processing

Frozen kinetochore samples were imaged on a 300 kV Titan Krios (ThermoFisher Scientific, MA) equipped with a high-brightness Field Emission Gun (x-FEG), a spherical aberration corrector, a GIF Bioquantum energy filter and a K3 direct electron detector (Gatan, Inc., CA). The spherical aberration coefficient of the objective lens on the microscope was reduced from an uncorrected 2.7 mm to ~0.01 mm. The K3 camera was operated in counted mode with a binning of 0.5 with dose fractionation enabled. Tilt series were collected using SerialEM^74^ dose-symmetric scheme with a tilt range of +/− 60°, a tilt step of 3°, and grouping of three images on either side (0°, 3°, 6°, 9°, −3°, −6°, −9° …). At each tilt, videos of 8 frames were acquired on the K3 camera. Data was acquired at two different magnifications with calibrated pixel size corresponding to 2.02 Å and 1.32 Å respectively. The total dose applied to each tilt series was 120 e^−^/ Å^2^ and the nominal defocus was set to −2.5 μm for each tilt after autofocusing on nearby area. Motion correction was performed for each video in Relion:SCR_016274^75^ to reduce motion-induced blurring effect. Motion-corrected micrographs were combined to form tilt series with the proper angular order using home-written script. Then the tilt series were automatically aligned by IMOD:SCR_003297 batchruntomo and reconstructed by tomo3d.^71,76^ The final tomogram reconstructions were post-processed by IsoNet to denoise and compensate missing wedge effect.^70^

#### Atomic force microscopy

Kinetochore samples were diluted in BRB80 (80 mM PIPES pH 6.8, I mM EGTA) buffer supplemented with 5 mM MgCl_2_ to concentrations varying between 30 and 80 mg/mL. Concentrations were optimized to create imaging fields with single, well-spaced kinetochores. For static imaging, approximately 20 μL of BRB80 + 5 mM MgCl_2_ buffer and 20 μL of kinetochore sample were deposited on a freshly cleaved mica surface and allowed to incubate for ~10 minutes. After incubation, 20 μL of buffer was added to a tip, and additional buffer was added to the mica surface accordingly to maintain a proper volume of liquid for imaging. To locate kinetochores of interest, a scan area of 4 × 4 μm^2^ was imaged. Areas that appeared to contain adequately spaced, single kinetochores, were then imaged using a scan area of 500 × 500 nm^2^ to further resolve kinetochore structure. For high-speed imaging, the following process was repeated, with volumes of 10 μL, rather than 20 μL.

All imaging was performed on the Asylum Cypher VRS AFM using tapping mode in liquid. Static images were acquired with a silicon BL-AC40TS tip (radius: 8 nm, resonance frequency: 110 kHz, spring constant: 0.09 N/m; Oxford Instruments, Abingdon UK). Before imaging, set point, scan lines, and scan rate were set to 500 mV, 256 × 256 pixels, and ~1.5 Hz, respectively. In liquid, the drive frequency of the tip was ~20 kHz. For high-speed imaging, a silicon USC-F1.2-k0.15–10 tip (radius: <10 nm, resonance frequency: 1.2 MHz, spring constant: 0.15 N/m, NanoAndMore, Watsonville CA) was used. Set point, scan lines, and scan rate were set to 250 mV, 256 × 256 pixels, and ~10 Hz, respectively. In liquid, the drive frequency of the tip varied between 450 and 550 kHz. Throughout both static and high-speed imaging, drive amplitude was maintained slightly above the point at which the tip started to contact the surface. Raw AFM data were processed using the Asylum Research (version 16.14.216) software. Static images, as well as images isolated from dynamic videos, were flattened using the magic mask feature. Height profiles were then created on the AFM height images.

#### Structural predictions

*K. marxianus* homologs were found by local sequence alignment searches as described above. ColabFold:SCR_025453^72^ was used to predict the structure of proteins which form a link between the inner and outer kinetochore (Cnn1, Ame1, Mif2). The full length of the *K. marxianus* proteins were used for structural predictions.

#### Statistical analysis, and figure generation

Statistical analysis was carried out with a variety of Python: SCR_008394 packages including: Lifelines for generation of rupture force survival curves and log-rank tests; Pandas for descriptive statistics of negative stain and cryo-ET measurement, microtubule binding, and gold labeling data. Optical trapping survival curves were generated in Python with Lifelines. Graphs for the proportion of doublets and singlets in negative stain, kinetochore microtubule interactions, and gold labeling were generated in python using Seaborn. Tomography distance boxplots were created in Python:SCR_008394 using Matplotlib. Scatter plots were generated in R with ggplot2. Gel images were cropped in Adobe Illustrator (Adobe, San Jose CA). Figures of protein structural models and alignments were generated in Chimera:SCR_004097 (University of California, San Francisco). Kinetochore cartoons were generated in Adobe Illustrator (Adobe, San Jose CA).

### QUANTIFICATION AND STATISTICAL ANALYSIS

All quantification and statistical analyses are indicated in the legend of their corresponding figures, along with sample sizes, tests used, and significance values. A p value of ≤ 0.05 was used as the threshold for significance unless otherwise indicated. Further details can be found in the method details section.

## Supplementary Material

supplemental movie 2

supplemental movie 1

supplemental movie 3

supplemental movie 4

supplemental items

supplemental data 1

supplemental data 2

supplemental data 3

supplemental data 4

supplemental data 5

## Figures and Tables

**KEY RESOURCES TABLE:** 

REAGENT or RESOURCE	SOURCE	IDENTIFIER

Antibodies		

M3DK	This study	N/A
Okp1 (*K. marxianus*)	This study	N/A
Nnf1 (*K. marxianus*)	This study	N/A
Spc24 (*K. marxianus*)	This study	N/A
Biotinylated His Tag	R&D Systems	Cat# BAM050; RRID: AB_356845
V5	Thermo Fisher Scientific	Cat# R96025; RRID: AB_2556564

Chemicals, peptides, and recombinant proteins		

Taxol	Sigma-Aldrich	Cat# T7402-25MG
Benzonase	Sigma-Aldrich	Cat# E1014-25KU
M3DK peptide	This study	N/A

Experimental models: Organisms/strains		

*S. cerevisiae* strain SBY8253 (W303 background):*DSN1-6xHis-3xM3DK:URA3*	Akiyoshi et al.^16^	N/A
*K. marxianus* strain SBY17411	USDA ARS Culture Collection	NRRL Y-8281
*K. marxianus* strain SBY18150 (SBY17411 background):*DSN1-6xHis-3xM3DK:KanMX*	This study	N/A
*K. marxianus* strain SBY21752 (SBY17411 background):*NDC80-6xHis-3xV5:NatMX, DSN1 -6xHis-3xM3DK:KanMX*	This study	N/A

Recombinant DNA		

Plasmid pSB2951 used to generate SBY18150	This study	N/A
Plasmid pSB3420 used to generate SBY21752	This study	N/A

Software and algorithms		

Igor Pro	Wavemetrics	https://www.wavemetrics.com/; RRID: SCR_000325
LabVIEW	National Instruments	https://www.ni.com/; RRID: SCR_014325
IMOD	Mastronarde & Held^70^	http://bio3d.colorado.edu/imod; RRID: SCR_003297
RELION	Scheres^71^	http://www2.mrc-lmb.cam.ac.uk/relion; RRID: SCR_016274
Python	Python software foundation	http://www.python.org/; RRID: SCR_008394
R	R Project for Statistical Computing	http://www.r-project.org/; RRID: SCR_001905
ColabFold	Miridita et al.^72^	RRID: SCR_025453
UCSF Chimera	Pettersen et al.^73^	https://www.cgl.ucsf.edu/chimera/; RRID: SCR_004097

Other		

50 nm Magnetic Nanobeads	CD Bioparticles	Cat# WHM-X047
300 mesh Quantifoil R 2/1 copper grids	Electron Microscopy Sciences	Cat# Q325CR1-2nm
10 nm gold particles for immunolabeling	Abcam	Cat# Ab201808

## Highlights

- Purification of relatively complete native *K. marxianus* kinetochores
- Native *K. marxianus* kinetochores sustain microtubule attachment under 9 pN force
- Cryotomography of *K. marxianus* kinetochores reveals a brush-like tri-laminar structure
- AFM on *K. marxianus* kinetochores confirms architecture and flexibility
