# Supplementary material for: Architecture of native kinetochores revealed by structural studies utilizing a thermophilic yeast
Source: Curr Biol. Author manuscript; Available in PMC 2024 Sep 11. (PMC11387133; doi:10.1016/j.cub.2024.07.036)
Supplement: supplemental items [file NIHMS2018420-supplement-supplemental_items.pdf]

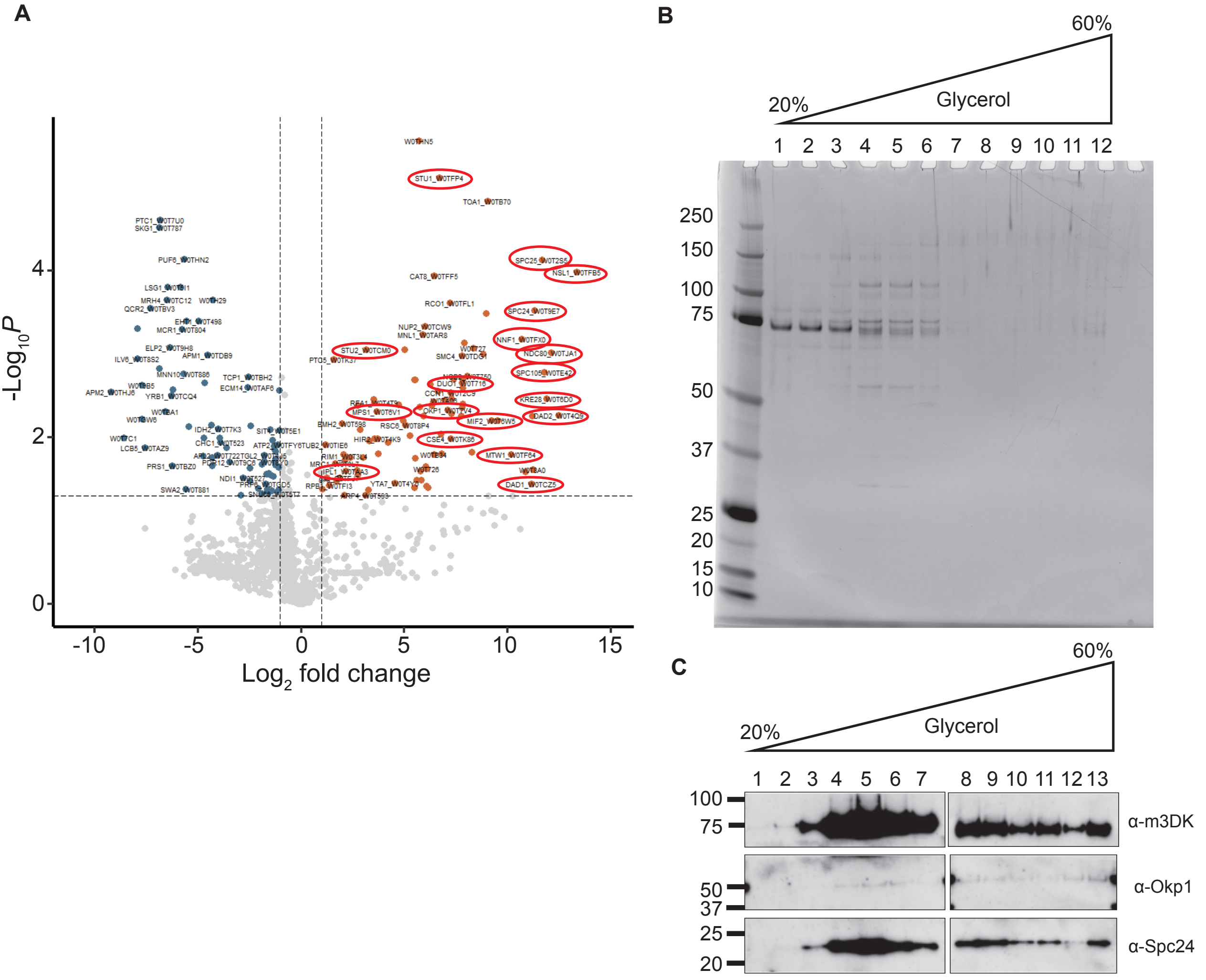

Supplemental Figure 1. Kinetochores components coelute as a single complex. Related to Figure 1 and Supplemental Table 1. A) Volcano plot comparing proteins enriched via mass spectrometry in Dsn1-M3DK purifications (SBY18150) compared to an untagged control (SBY17411). Orange dots towards the top right indicate significant upregulation while blue dots to the top left indicate significant down regulation. Dashed lines indicate a significance cut off of p = 0.05 and fold change of 2x. Kinetochore and kinetochore associated proteins have been highlighted with red ovals. B) Kinetochores coelute through a glycerol gradient. 100 µl fractions were collected starting from the top (20% glycerol) to the bottom (60% glycerol) and protein content visualized by silver stain gel. C) Samples prepared identically to from B were used for immunoblotting, probing for Dsn1 through the m3DK tag (top), Okp1 (middle), or Spc24 (bottom). All samples run in C are frome the same biological replicate, however fractions 1-7 and 8-13 were run on sepatate gels to accommodate the necessary lanes. In both B and C free protein can be seen in lanes 2-7, while more complete kinetochores can be seen coeluting at lower levels in lanes 10 and up.

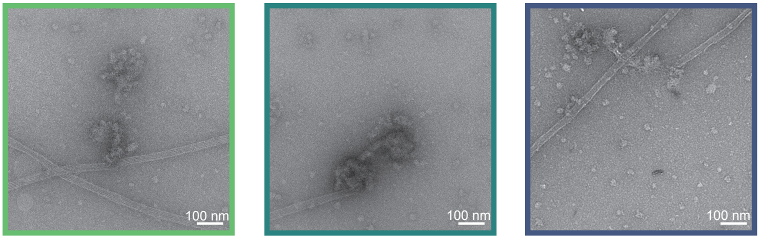

Supplemental Figure 2. Kinetochores bind to both the lattice and tips of microtubules. Related to Figure 3. Representative images of kinetochores interacting with microtubules on either the lattice (left), tip (center), or both (right). Scale bars are 100 nm.

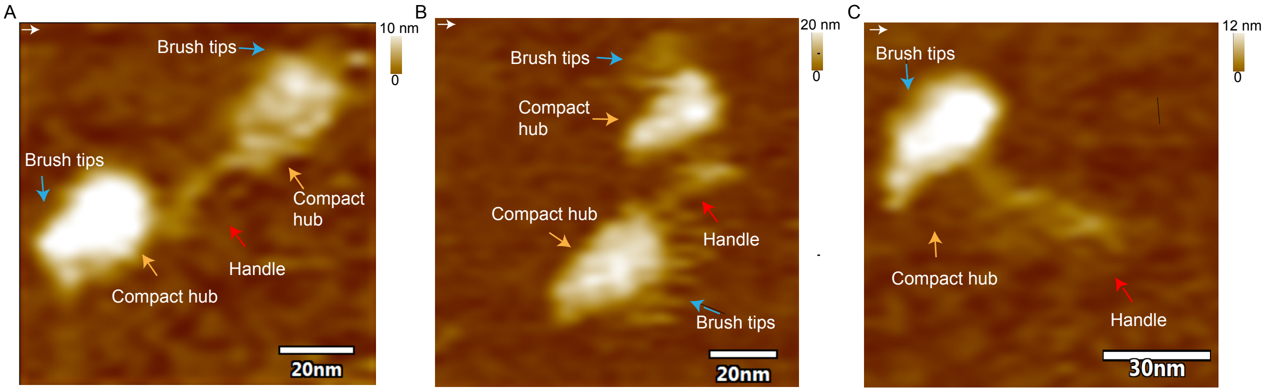

Supplemental Figure 3. Doublet kinetochores can be seen by AFM. Related to Figure 5. Three examples of AFM height images of doublet kinetochores. The blue arrows indicates the brush tips, the orange arrows indicates the compact hub, and the red arrow indicates the brush handle. The x-y scale bar is 20 nm. The scanning rate is 1 s/frame with 256 x 256 pixels. White arrows at the top left of the images indicate the scanning direction. The z-scale is 0 to 20 nm (dark to light brown).

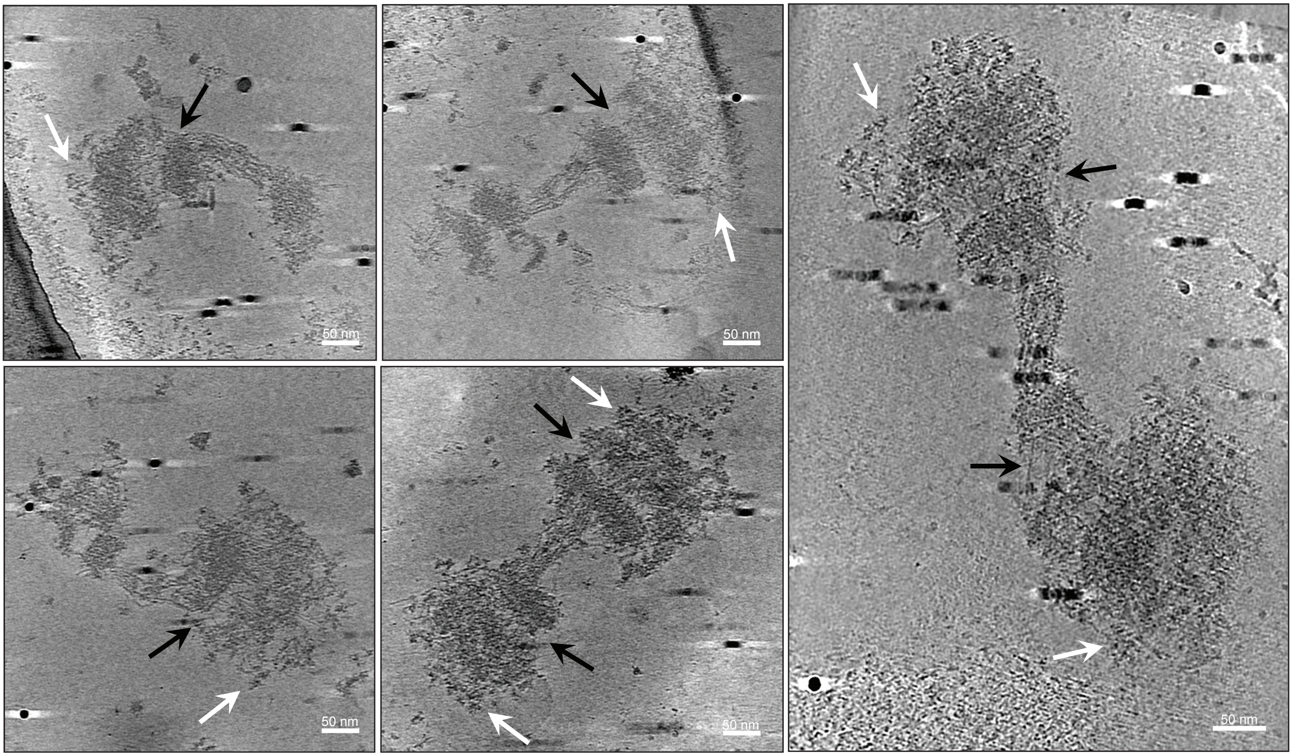

Supplemental Figure 4. Additional views of kinetochore features. Related to Figure 6. Gallery of kinetochores revealed by cryo-electron tomography. Black arrows: flexible fibrils connecting the brush tips to the compact hub. White arrows: brush tip extensions. Scale bars are 50 nm in each panel.

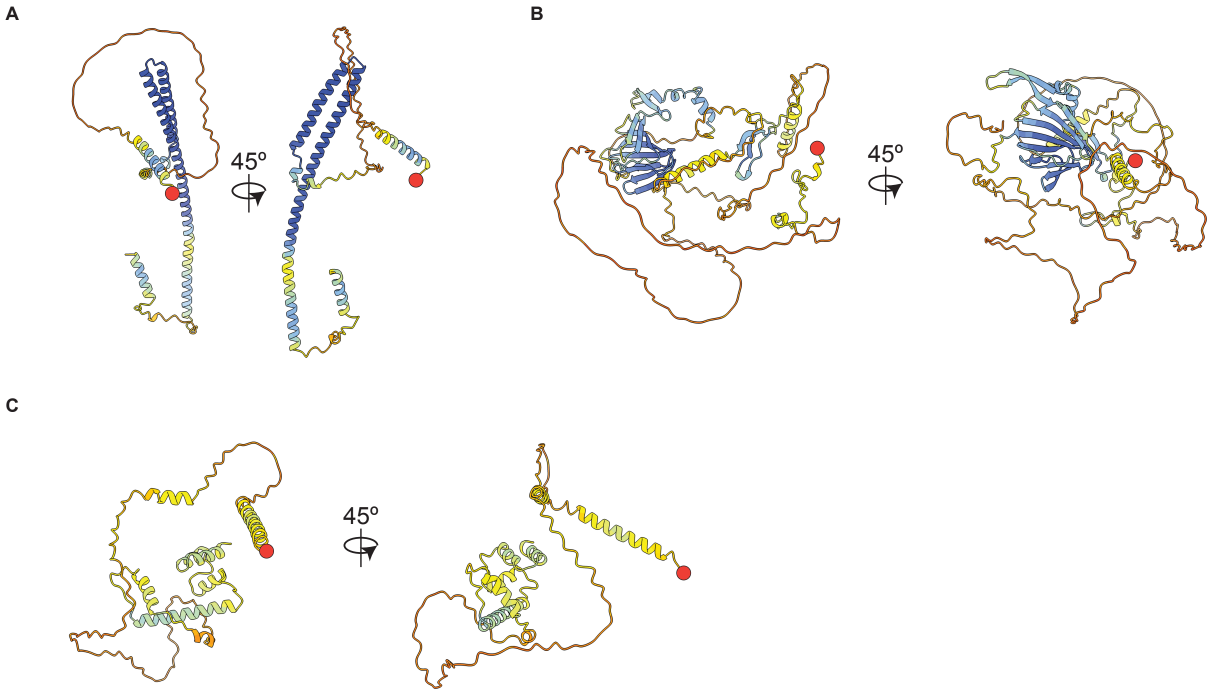

Supplemental Figure 5. Models suggest high flexibility in the links between the inner and outer kinetochore. Related to Figures 6 and 7. Alphafold ColabFold predictions of Ame1, Mif2, and Cnn1 show long disordered tails. A) A structural prediction for *K. marxianus* Ame1 with its N-terminus marked with a red circle. B) A structural prediction for *K. marxianus* Mif2 with its N-terminus marked with a red circle. C) A structural prediction for the potential *K. marxianus* Cnn1 homolog with its N-terminus marked with a red circle. All models are colored by per-residue confidence score (pLDDT) where blue indicates high confidence and red indicates low confidence.

Complex	Protein	*S. cerevisiae* molecular weight (kDa)	Expected molecular weight of *K. marxianus* homolog (kDa)	*K. marxianus* protein sequence ID	Alignment score to *S. cerevisiae*	E. value
Ndc80c	Ndc80	80.5	78.4	XP_022677987.1	551	0.0E+00
	Nuf2	52.9	52.9	XP_022676124.1	393	2.0E-138
	Spc24	24.6	20.9	XP_022675266.1	47	2.0E-11
	Spc25	25.2	25.2	XP_022673631.1	89.4	1.0E-26
Spc105	Kre28	44.7	41.1	XP_022673577.1	60.5	1.0E-14
	Spc105	105	97	XP_022677423.1	184	3.0E-52
Dam1c	Hsk3	8.09	7.3	XP_022675267.1	44.3	4.0E-13
	Dam1	38.4	38.9	XP_022677914.1	158	3.0E-50
	Duo1	27.5	22.9	XP_022673782.1	94.4	2.0E-28
	Spc19	18.9	17.7	XP_022678124.1	80.1	2.0E-26
	Spc34	34.1	32.3	XP_022676083.1	177	5.0E-59
	Dad2	15.1	13.7	XP_022673936.1	97.1	6.0E-32
	Dad4	8.16	8.5	XP_022677868.1	102	3.0E-36
	Ask1	32	26.7	XP_022678310.1	113	1.0E-34
	Dad1	10.5	11.9	XP_022676704.1	99.8	6.0E-34
	Dad3	10.8	9.5	XP_022674564.1	79	4.0E-26
MIND	Nnf1	23.6	23.6	XP_022677716.1	140	7.0E-47
	Dsn1	65.7	63.2	XP_022676920.1	43.5	1.0E-08
	Nsl1	25.4	25.7	XP_022677830.1	144	6.0E-48
	Mtw1	33.2	33.4	XP_022676573.1	228	2.0E-78
Stu2	Stu2	101	97.9	XP_022677161.1	420	3.0E-137
Ctf19c	Iml3	28.1	-	-	-	-
	Chl4	52.7	45.3	KAG0682270.1	116	6.0E-34
	Nkp1	27	24.3	XP_022674906.1	36.2	2.0E-07
	Nkp2	17.9	17.6	BAP71547.1	19.6	1.4E-02
	Ame1	37.4	33	XP_022673771.1	64.3	3.0E-16
	Okp1	47.3	51.2	XP_022675341.1	118	7.0E-34
	Ctf3	84.3	71.9	XP_022677695.1	160	8.0E-31
	Mcm16	21.1	18.8	XP_022677702.1	42.7	3.0E-10
	Mcm21	43	34.8	XP_022675474.1	70.9	3.0E-18
	Mcm22	27.6	26.1	XP_022676344.1	69.7	4.0E-19
	Cnn1	41.3	30.2	QGN15715.1	-	-
	Wip1	10.2	10.2	XP_022674922.1	24.6	5.0E-05
	Mif2	62.4	60.9	XP_022675205.1	333	4.0E-112
Cbf3	Ndc10	112	88	XP_022674868.1	239	4.0E-71
	Cep3	71.4	72.2	XP_022674990.1	273	1.0E-87
	Ctf13	56.3	46	XP_022674481.1	129	1.0E-37
	Skp1	22.3	20.9	XP_022677187.1	282	4.0E-103
Cbf1	Cbf1	39.4	40.6	QGN15070.1	166	1.0E-52
Cse4	Cse4	26.8	22.4	XP_022678314.1	192	3.0E-67

Supplemental Table 1. A table of *S. cerevisae* kinetochore proteins and their homologs in *K. marxianus* identified by mass spectrometry. Related to Figure 1 and Supplemental Figure 1. Proteins which were identified in the mass spectrometry data are in white rows. Proteins which were not identified in the mass spectrometry but were found by BLAST search of NCBI taxID:4911 using the S. cerevisiae protein as a reference are colored green. Proteins which were found by BLAST search of NCBI TaxID: 4911 using Kluyveromyces lactis proteins as references are colored in blue. Proteins for which no homolog was found are colored red. Alignment scores and expected values are listed, where higher alignment scores and lower E. values indicate better matches.
